# New Human Follitropin Preparations: How Glycan Structural Differences May Affect Biochemical and Biological Function and Clinical Effect

**DOI:** 10.3389/fendo.2021.636038

**Published:** 2021-03-19

**Authors:** James A. Dias, Alfredo Ulloa-Aguirre

**Affiliations:** ^1^ Department of Biomedical Sciences, State University of New York at Albany, Albany, NY, United States; ^2^ Red de Apoyo a la Investigación, National University of Mexico-Instituto Nacional de Ciencias Médicas y Nutrición SZ., Mexico City, Mexico

**Keywords:** follitropin, glycoprotein, glycosylation, pharmocodynamics, pharmacokinetics, therapeutics

## Abstract

It is well accepted that pituitary follitropin is secreted into the circulation as a mixture of variants, which differ not in primary structure but rather at the level of glycosylation. These glycosidic forms vary in the number of glycosylation sites filled, complexity of glycosidic chains, and sialylation and sulfation. It is generally agreed that high sialylation, 2,3 sialic acid capping of terminal N-acetyl galactosamine or galactose leads to longer circulating half-life, by blocking binding of asialoglycoprotein receptor (ASGPR) in the liver. In contrast, 2,6 sialic acid found in humans does not prevent recognition of galactose and N-acetyl galactosamine by ASGPR. Few studies on clinical outcomes comparing differences in sialylation of follitropin found in commercially available preparations are available. Thus, there is a clear need for a consortium of open data to address this unmet need. Recently, FSH glycosylation, primarily on the β-subunit, which varies as women age, has emerged as a key modifier of follitropin action, with profound biological effects *in vivo* in animal models. To date, limited information of recombinant follitropin hormone preparations is available. Thus, most of the studies with FSH that is well characterized biochemically have been done *in vitro*, with engineered non gonadal host cells bearing recombinant receptors or in animal models. Since limited studies in human granulosa cells are available, a question is whether structural differences in glycosylation in commercially available follitropin affects biological function and clinical effect in humans. The presence of fucose, for example, has not been studied greatly even though, in the case of antibody therapy it has been shown to have a large effect on antibody targeting. This review on glycosidic variability of follitropin from the biochemical/structural point of view reflects on this question and presents an assessment in the context of available published data. If clinical differences are to be expected or not, the readers will have a better understanding of the evidence for and limitations of such expectations.

## Introduction

In this review, the gonadotropin follitropin is discussed exclusively in the context of how the carbohydrate structures modulate biochemical activity and pharmacodynamics. Moreover, it is an objective of this review that consideration should be given to how the nature of FSH carbohydrate complexity may impact the quaternary and tertiary structure of the heterodimeric gonadotropin molecule and how it may ultimately affect its biological activity from a pharmacodynamic perspective as well as from well accepted information on pharmacokinetics impacts. The glycoprotein hormone follicle stimulating hormone (follitropin, FSH), is used clinically in men to induce spermatogenesis (1) and in women to induce ovarian follicle growth and promote maturation to a preovulatory follicle containing a fertilization competent oocyte (2). FSH is produced in the pituitary gland as two subunits, one subunit common to all glycoprotein hormones, the α-subunit, and another subunit specific for each hormone, the β-subunit. The addition of carbohydrate to the protein backbone (glycosylation) occurs as the protein is being made. Subunits combine following folding of the individual subunits to form the heterodimeric active molecule. The subunits each contain two potential glycosylation sites. In postmenopausal women pituitary secretion of follitropin is very high due to decreasing estrogen levels. So high in fact, that prior to the advent of recombinant DNA technology, the therapeutic form of follitropin was purified from the urine of post-menopausal women using standard biochemical methods amenable to crude starting materials (3). These preparations of human menopausal gonadotropin (hMG) generically referred to as urofollitropin, proved useful in the clinic (**Table 1**). However, the purity and heterogeneity of purified urofollitropin (HP-hMG) was still a concern. Follitropin in urine differed from naturally occurring pituitary follitropin in degree and complexity of their glycans and contamination with a related glycoprotein hormone (luteinizing hormone) and other proteins (**Table 1**). Although highly purified versions of urofollitropin (HP-hFSH) became available with minor amounts of luteinizing hormone, the heterogeneity of gonadotropin within these preparations was still an issue separate from their purity, the former of which has been improved by using advanced methods including monoclonal antibody affinity purification (3).

**Table 1:** Naturally occurring and commercially available *recombinant* follitropin preparations and nomenclature.

Generic Name​	Brand name examples​	Characteristics​
Urofollitropin (Fertinex)​	Fertinex (HP-FSH) immunoaffinity (8.5–13.5 IU/ug) Metrodin​ (87 IU/mg) (discontinued in US) Bravelle (2%LH)(discontinued in US), Menopure, Ferring (available in US)	From post-menopausal urine; more acidic forms than recombinant FSH. Isoelectric point (pI <4.0, 40% of preparation and higher than 4.0 (74%) in highly purified preparations); both α2,3 and α2,6 sialylation) and reduced sulfated glycans compared to pituitary FSH, less core (~23.9%) fucose and less bisecting glycans than pituitary FSH.​
Pituitary FSH​	Not commercially used​	Derived from human pituitary gland; both α2,3 and α2,6 sialylation and with sulfated glycans, 44–53% core fucose.​
Follitropin alpha​	Gonal-F^®^ EMD Serono of Merck KGaA,Germany​ (13.6 IU/ug) Bemfola^®^ Finox Biotech, Sweden (US rights obtained by Gedeon Richter Plc.​ Ovaleap^®^ Teva Pharma, Israel ​ Cinnal-f^®^Cinnagen, Iran​	Derived through recombinant DNA technology and expressed in Chinese hamster ovary (CHO) cells. Isoelectric point (pI = 4–5) 91% >4.0 so profile more basic than urinary FSH, with only α2,3 linked sialic acid​, 30-36% core fucose. Only Gonal F is currently available in US.
Follitropin beta​	Follistim^®^ Organon,​ (available in US) Puregon^®^ Merck​	Derived through recombinant DNA technology and expressed in Chinese hamster ovary (CHO) cells. Isoelectric point (pI = 3.5–5.5) profile more acidic than follitropin alpha, α2,3 linked sialic acid.; 13.7% core fucose.
Follitropin delta​	Rekovelle^®^ Ferring, Switzerland​	Derived through recombinant DNA technology and expressed in **human** cells. Contains both α2,3 and α2,6 sialylation patterns.
Follitropin epsilon​	Glyco Express Glycotope, Berlin​	Derived through recombinant technology and expressed in human cells. Similar as above but with some differences.​

Naturally occurring follitropin from human pituitaries is not used commercially but has been the gold standard for many biochemical and biological studies as well as a comparator for recombinant follitropin. Urinary follitropin is available commercially. Recombinant follitropin Gonal-F^®^, and biosimilars Bemfola^®^, Ovaleap^®^ and Cinnal-f^®^ are grouped into one class called follitropin Alpha. The follitropin Beta class includes Follistim^®^ and Puregon^®^. Follitropin Delta included the follitropin Rekovelle^®^ which is produced using a human cell line and results in a product that possesses sialic acid2,6 as well as sialic acid2,3 capped Gal. Follitropin Epsilon is also produced in a human cell line but is in a different group because it is claimed to have different glycosylation to Rekovelle^®^ and it is not yet commercially available. Isolelectric profile as reported in (4) and fucose as reported in (5).

**Figure 1:**
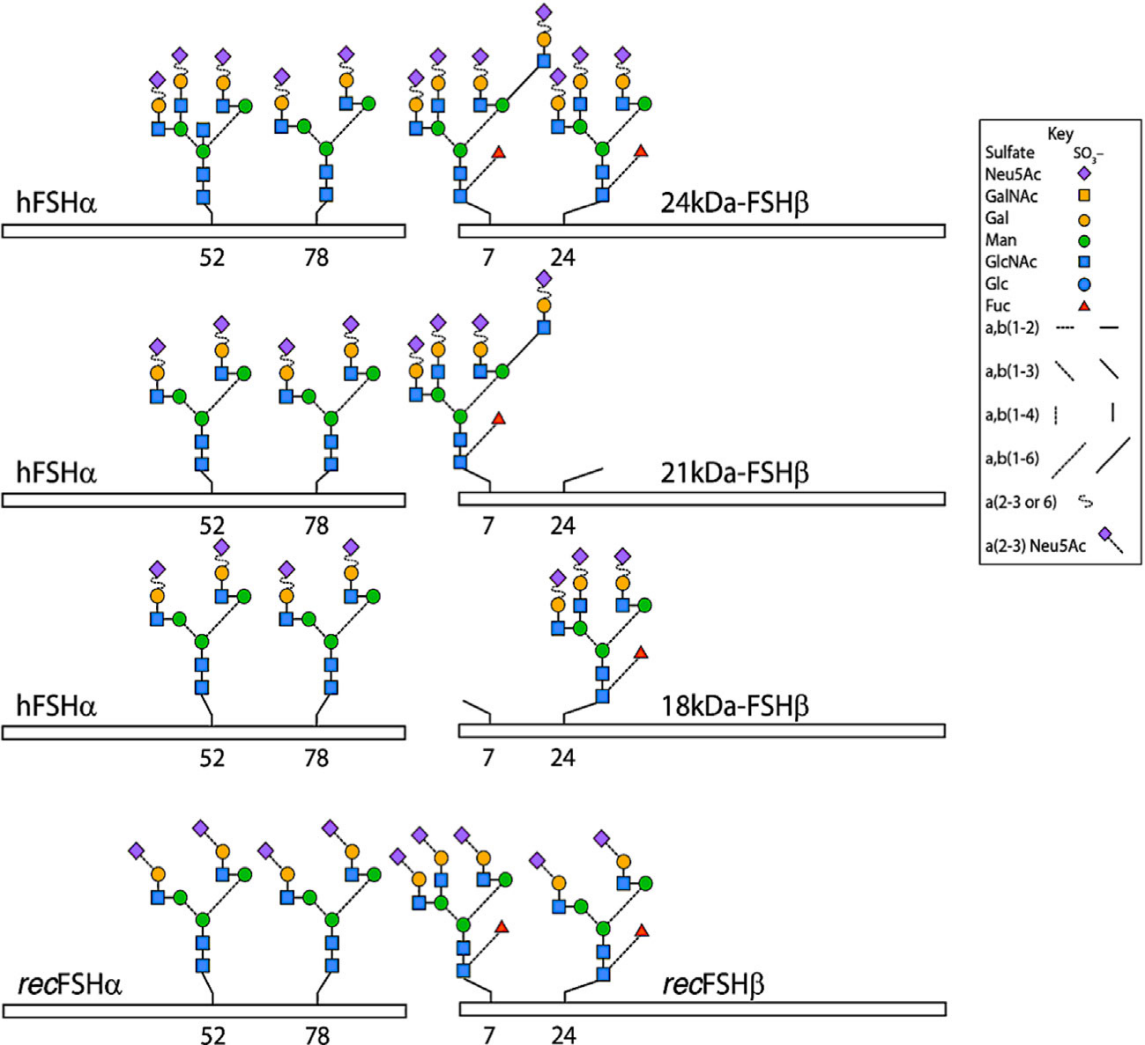
Typical glycans attached to human pituitary FSH and human recombinant FSH produced by Chinese hamster ovary cells (*rec*FSH) glycoforms. The bars indicate the common-FSH α and hormone-specific FSH β-subunits. N-glycosylation sites are indicated by the numbers below the bars. Note that glycans at the α−subunit of FSH^18/21^ are in fact smaller (*i.e.* biantennary) than those present in FSH^24^. Modified from (9), with permission.

As stated previously, it is well accepted that pituitary follitropin is secreted into the circulation as a mixture of glycoform variants; these differ not in protein primary structure (amino acid sequence) but rather at the level of glycosylation. N-linked glycosylation occurs at the asparagine (Asn, N) amino acid when it is positioned in a glycosylation sequon (Asn-X-Ser/Thr, where X can be any amino acid followed by either serine or threonine). N-linked glycosylation determines pharmacokinetics or half-life of follitropin and its biological effects (6, 7). The heterogeneity of gonadotropins stems largely from their variable N-linked carbohydrate composition and glycans (building blocks at each N-linked site) complexity (7, 8) as seen in **Figure 1**. The building block N-acetyl glucosamine (GlcNAc) is linked to asparagine followed by the addition of another GlcNAc, then by one to three Mannose residues that can branch into 1, 2 or 3 antennae. The antennae are then extended by GlcNAc and galactose, the latter of which can be capped by N-acetyl neuraminic acid (sialic acid) which imparts a positive charge on each antenna.

**Figure 2:**
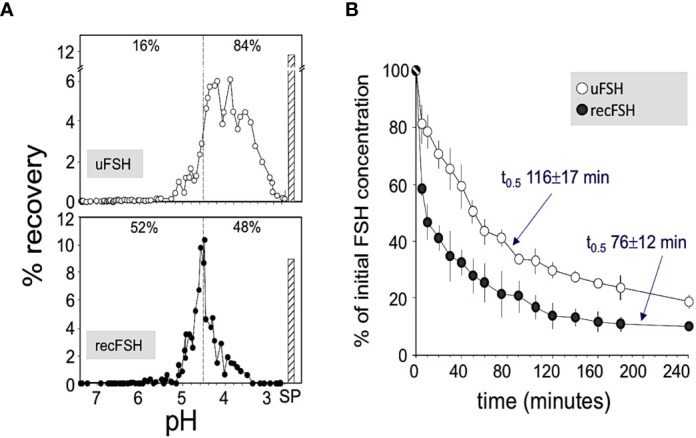
**(A)** Representative patterns of pH distribution of immunoreactive FSH after chromatofocusing of highly purified urinary FSH (upper panel; Fertinorm HP, formerly distributed by Serono, Switzerland) and recombinant follitropin beta produced by CHO cells (lower panel; Puregon, Organon International BV, Oss, The Netherlands). Vertical broken lines separate charge isoforms with pH values >4.5 and <4.5. The percent recoveries within each pH window are indicated at the top of each pattern. SP, salt peak. **(B)** Plasma disappearance curves of urinary FSH (uFSH) or recombinant FSH produced in CHO cells, from rat circulation. Approximately 60 µg of immunoreactive uFSH or recombinant FSH were injected i.v. and blood samples were obtained at 5 and 10 min after the injection and thereafter at regular time intervals during the ensuing 4 h. Samples were analyzed for FSH content by a specific human FSH fluoroimmunoassay. Values are mean ± SEM of three to five independent studies. Reproduced from (10) with permission.

**Figure 3:**
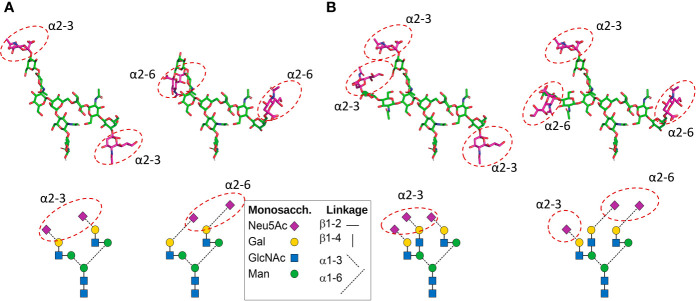
Positions of Neu5Ac residues in CHO cell-produced recombinant FSH **(A)** and pituitary FSH glycans **(B)**. Top: 3D models show neutral monosaccharide carbon atoms in green. The sialic acid residue carbon atoms are shown in magenta, like the symbols in the glycan diagrams below each 3D model. The 3D models were built using the current Glycam web tool (http://glycam.org). **(A)** In Gonal-F^®^ and Bemfola^®^, >90% of glycans at αAsn52, αAsn78, and βAsn24 are biantennary with two α2–3 linked Neu5Ac residues (the only significant variant has one Neu5Ac instead of two). The position of potential α2–6 Neu5Ac residues (which are absent in CHO cell-produced recombinant FSH preparations) in the biantennary structure shown at the *right* is also shown. **(B)** In pituitary FSH the most abundant types of glycan are the triantennary type. Two alternatives exist (11): *a.* all three Neu5Ac residues can be linked α2–3; *b.* alternatively, there can be 2 linked α2–6 and one linked α2–3. The one α2–3 linked Neu5Ac on the Galβ1-4GlcNAcβ1-2Manα1-3 branch (*right*) was arbitrarily placed. A diagram of each glycan is shown below to help ID the glycans. Figure courtesy of Dr. George R. Bousfield, Department of Biological Sciences, Wichita State University, Wichita, KS, USA.

These glycosidic forms exist as *isoforms* and *glycoforms*. FSH glycoforms may have the same net charge but can differ structurally in two ways: 1. The complexity and variation of carbohydrate structures at each of the four potential attachment sites (ie. bi-, tri- or tetra-antennary) and 2. the presence or absence of glycosylation at the N-residue in any glycosylation sequon (5, 7) of the β-subunit, as represented in **Figure 1** and **Table 2**. Isoforms of FSH are charge variants generally detected by isoelectric focusing (**Figure 2**) and separated based on net glycoprotein charge, which to a large extent is determined by sialic acid content as visualized in **Figures 2** and **3**. Here it is important to emphasize that human FSH α-subunit is always glycosylated on both glycosylation sequons (Asn52 and Asn78).

**Table 2:** Examples of how net charge may not predict follitropin pharmacokinetics which is determined by both completeness of sialylation (per molecule) as well as number of potential sialylation sites (Gal) per molecule of follitropin.

Example	N-linked sites	Branches	Gal sites	Sialic acid(Capped Gal)	Uncapped Gal	t^1/2^ Impact
A	4	biantennary	8	8	0	slowest
B	4	biantennary	8	0	8	fastest
C	4	triantennary	12	6	6	faster
D	4	triantennary	12	8	4	fast
E	4	triantennary	12	11	1	slow

Follitropin preparations that contain a high proportion of isoforms with a high content of sialic acid capping of galactose have a low isoelectric point (ie. <4.0). As such at pH 7.0 as in the circulation, those isoforms are negatively charged. These isoforms are also are less likely to be recognized and cleared by the asialoglycoprotein receptor (ASGPR) because alpha-2,3 sialylation of galactose (capping) dissuades interaction with the ASGPR).

## Biosimilars Current Status

The advent of molecular biology and recombinant DNA technology allowed for *de novo* synthesis of therapeutic proteins *in vitro*. Therapeutic proteins could now be produced in large scale cell culture using well defined media and bioreactors. Recombinant technology further improved the purification process by decreasing the complexity of the starting material. Readily available and more predictable large-scale production of follitropin was possible.

For many years, follitropin produced by recombinant DNA methods was used clinically without regard to glycosylation status. A first generation of follitropin was referred to as follitropin alpha (12) (**Table 1**). Another clinical preparation of recombinant follitropin also prepared by recombinant DNA methods was generically labeled follitropin beta (13). Follitropin alpha was more acidic (pI 5.69–3.21) than follitropin beta and urofollitropin (PI 5.58–3.8) but more like pituitary FSH. Yet the expression plasmids, selection methods and cell lines used for expression as well as the purification process itself, which included monoclonal antibody affinity chromatography may have produced glycosylation differences between follitropin alpha and follitropin beta. For the most part, glycosylation is similar between follitropin alpha and follitropin beta preparations. However follitropin alpha appeared to contain more tetra-antennary structures than follitropin beta (12, 14). This would have the net effect of potentially creating more opportunities for sialylation and increasing the net positive charge per protein molecule compared to follitropin beta, and as a result, may affect their relative pharmacokinetics and their relative *in vivo* bioactivities or pharmacodynamics. In this regard there is some evidence that follitropin beta induced a greater estradiol response in patients whereas follitropin alpha was associated with a higher pregnancy rate (15).

The Patient Protection and Affordable Care Act (PPAC Act), signed into law by USA President Obama on March 23, 2010, amended the Public Health Service Act (PHS Act) to create an abbreviated approval pathway for biological products that are demonstrated to be “highly similar” (biosimilar) to or “interchangeable” with an FDA-approved biological product (comparator) (16). While there are two biosimilar follitropin preparations, Bemfola^®^ and Ovaleap^®^, which have been approved by the European Medicines Agency (EMA) (**Table 1**), neither have been approved for use in the US and neither are currently on the market, awaiting further clinical and in some cases pre-clinical studies.

Under the US framework, to meet the higher standard of interchangeability, a sponsor must demonstrate that the biosimilar product, for example Bemfola^®^ and Ovaleap^®^ can be expected to produce the same clinical result as the reference product, in this case Gonal-F^®^ (also known as follitropin alpha). This clinical similarity must be achieved in any given patient and, for a biological product that is administered more than once, that the risk of alternating or switching between the biosimilar product and the reference product is not greater than the risk of maintaining the patient on the reference product. Interchangeable products may be substituted for the reference product by a pharmacist without the intervention of the prescribing health care provider. Whereas the EMA carries out the scientific review of a biosimilar, the evaluations do not include recommendations on whether the biosimilar is interchangeable with the reference medicine, and thus whether the reference medicine can be switched or substituted with the biosimilar. Ultimately, the decision whether to allow interchangeable use and substitution of the reference biological medicine and the biosimilar is made at the national level.

One other consideration is the need to distinguish between a biosimilar and a form of FSH which significantly differs in properties of the “originator” comparator or in primary structure. Clearly, FSH biosimilars will possess a primary structure (amino acid sequence) of each of the α- and β-FSH subunits that are identical regardless of the recombinant FSH products. However, the expressing cell line and production processes can influence the structural characteristics of glycans in the recombinant FSH, with differences in glycosylation profile, sialic acid pattern, and fucosylation. Biosimilar recombinant human follitropin Bemfola^®^ was compared with its reference medicinal product Gonal-F^®^. Both are produced in Chinese hamster ovary (CHO) cells. Mass spectrometry analysis revealed differences in glycosylation complexity at asparagine 52 (Asn52, N52). This glycosylation site of the α-subunit of FSH, plays a pivotal role in the interface of FSH with the FSH receptor (17). Bemfola^®^ exhibited a lower proportion of bi-antennary structures [~53% vs ~77%] and a higher proportion of tri-antennary [~41% vs ~23%] and tetra-antennary structures [~5% vs <1%]) than Gonal-F^®^ (5, 18). At Asn52, nearly all glycans were present as α-fucosylated complexes in both Bemfola^®^ and Gonal-F^®^ but at a ratio of 1:2 (18). These structural differences appeared to play a greater role in pharmacokinetics than in pharmacodynamics of the early signal transduction pathways. Differences in core fucosylation were observed when comparing the Asn52 glycan GlcNac1 position of Gonal-F^®^ to Bemfola^®^. As noted above Asn52 on follitropin alpha subunit is most clearly associated with the hormone-receptor interface and biological activity (17, 19). Typically, it is difficult to observe well defined glycan structures on glycoproteins. However, when core fucosylation is seen in well-defined N-glycan structures it is in most cases attributed to carbohydrate-protein and/or carbohydrate-carbohydrate interactions. These interactions which have been observed in immunoglobulin crystal structures may function as “molecular glue” to help stabilize inter- and intra-molecular interactions (20). Interestingly, those preparation-specific glycosylation differences between Gonal-F^®^, and Bemfola^®^ observed using mass spectrometry (18) did not affect cAMP signaling and β-arrestin recruitment when the two preparations were compared (21). Nor was any difference in FSH induced progesterone and estrogen synthesis observed when tested in primary cultures of human granulosa cells (21). On the other hand, in this particular study cell treatment by Gonal-F^®^ induced a rapid intracellular Ca2+ increase, which was about 230-fold higher than vehicle and occurred within 1 to 2 s after hormone addition. Bemfola^®^ and another biosimilar, Ovaleap^®^ induced only a minimal, not significant intracellular Ca2+ increase (21). Therefore, an ancillary question remains whether differences may exist in gene expression and phosphoproteome profiles reflecting signaling downstream from Ca2+ release.

In addition to biosimilars, genetic manipulation of the primary sequence has created new forms of follitropin. For example, additional glycosylation sequons have been engineered to increase glycosylation attempting to increase blood half-life (22–24). Genetic engineering of a long carboxy-terminal extension containing O-glycosylation sites has been successful in creating forms of follitropin with prolonged half-life, now clinically available in Europe but sadly not in the United States. That long-acting form of follitropin obviates multiple injections of follitropin in some clinical situations (25–28). In addition, coupling of follitropin to functional domains such as the Fc binding domain of antibodies has facilitated the development of follitropin therapeutic prototypes which can be inhaled as micronized gonadotropins that cross the endothelium and enter the bloodstream (29). Finally genetically engineered amino acid substitutions have produced super-agonists of gonadotropins (30). For the purposes of this review, it will be assumed that amino acid sequences of follitropin subunits are identical to the naturally occurring or wild-type (WT) subunits regardless of the recombinant follitropin products unless otherwise specified and only human follitropin preparations will be considered.

## Structural Aspects of Follitropin which Affect *Pharmacokinetics*

*Glycosylation:* Naturally occurring human pituitary follitropin evidences high glycosylation macroheterogeneity. This occurs exclusively at potential N-glycosylation sites on the β-subunit, which are filled before the heterodimeric protein is secreted into the blood (8). These forms of follitropin are referred to as *macro heterogeneous* follitropin *glycoforms*, which result from the absence of one or more oligosaccharides structures from a given hormone variant (**Figure 1**) (6). For the purpose of this review, we will use the term *glycoform* to refer only to the forms of FSH varying in the number of glycans attached to the beta subunit. In the next section the impact of glycoform variation on FSH activity will be covered. Then in the section on isoforms which follows, the impact of sialylation on FSH activity will be discussed in terms of pharmacokinetics. In addition, the interplay between glycoforms and isoforms will be discussed as it relates to the net activity of FSH particularly at the level of the receptors where desialylation can increase affinity of follitropin for its receptor. In addition absence of carbohydrate at the follitropin beta subunit increases affinity and imparts enhanced activity at the level of the receptor.

*Glycoforms:* Glycoforms of follitropin can have different biological half-lives due to differences in clearance (31). Physiologically, FSH secreted from human pituitaries is fully glycosylated on the alpha subunit but varies in glycosylation at the beta subunit (5). Engineered glycosylation mutants of human FSH showed more rapid clearance when tested in rats (6) and had different pharmacodynamic properties owing to the mixtures of different glycoforms. For human follitropin the beta subunit residues appear to have the greatest effect on clearance in rats (6). However, studies in murine animal models must be verified clinically, because naturally occurring glycoforms of follitropin with fewer filled glycosylation sites at the beta subunit have been shown to bind to receptor more rapidly, and are more active at the target cell level in both human granulosa cells and HEK293 cells than fully glycosylated follitropin (with all four sites filled) (32). Indeed, recombinant beta FSH glycosylation variants can rescue beta FSH knockout animals (33). Depending on the outcome desired, it may not be ideal that the final product exhibit the same half-life as fully glycosylated follitropin (10, 34). For example, a longer half-life may be more appealing to women if it means fewer injections, while a patient prone to OHSS may be more suited to a shorter half-life gonadotropin with better temporal control as with the beta FSH threonine 26 sequon null mutant.

The production of different ratios of FSH *glycoforms* in the pituitary gland will be recapitulated in the process of manufacturing. However, to produce a more uniform FSH preparation than the one naturally produced by the pituitary is a challenging problem for glycoprotein hormones. This is because the two beta subunit glycosylation sites are not filled consistently (8, 35–37) (**Figure 1**). Therefore, from a biopharmaceutical perspective, production of a “uniform” product will require the consistent production of the same *ratio* of *glycoforms* with each batch over the lifetime of the product. The ratio of fully glycosylated versus partially glycosylated follitropin should be a further consideration when assessing whether preparations are similar or different, and whether these differences affect the pharmacodynamics and the net clinical response.

*Isoforms:* Historically, the net charge of follitropin preparations has been measured by isoelectric focusing or high resolution chromatofocusing. The separation of follitropin in a preparation in either the acidic or basic fractions has been taken as an indicator of the degree to which follitropin in a preparation is sialylated and sulfated (38, 39). This has led to a nomenclature of follitropin charge (basic or acidic) *isoforms* (38). Moreover, the pharmacokinetics of a follitropin preparation was attributed to its net charge. Thus follitropin isoforms of basic charge have been shown to have a faster clearance rate than the more acidic follitropin isoforms (higher sialic acid content) (10, 40) (**Figure 2** and **Table 2**). In theory and to a great extent in practice, a follitropin preparation with a less acidic isoelectric point profile should not present problems in administering the correct dose, since the doses are adjusted according to individual patient response in assisted reproductive technologies (ART) therapy. However, it is now appreciated that the net charge of a follitropin preparation can be representative of the glycoform profile as well as the degree of sialylation. Desialylation of follitropin decreases both the net charge and plasma half-life (41). Therefore, heavily sialylated follitropin should result in a slower clearance and therefore a greater efficacy when compared to a less sialylated form of follitropin when it is compared by mass (protein content) and not by bioassay units (International Units, IU) (**Figure 2**). Despite its purity, follitropin alpha dosing was originally vialed based on *in vivo* bioactivity measured in a rat bioassay against a crude international reference preparation, hence the potency was expressed as International Units (IU). The variability of this assay was quite high. In 2005, Gonal-F^®^ was available “filled by mass” (42). This really took advantage of the purity of the preparation and produced a more consistent therapeutic effect that was no longer dependent on the rat bioassay. That approach was achievable because the batch-to-batch variation was low with regard to the carbohydrate content and degree of sialylation, which will be discussed below in terms of its effect on *in vivo* biopotency. The mass of FSH was based on the protein content of the preparation (43). Nevertheless, the use of mass rather than *in vivo* bioactivity can still lead to differences in efficacy between new preparations of follitropin, if their glycoform or isoform profiles are different as will be discussed. To understand why this may be so, in the case of isoform profile, one must identify how sialylation prolongs half-life as discussed next.

As early as 1970 it was recognized that desialylation of the glycoprotein hormones increased their rate of clearance primarily through a hepatic pathway (43). N-Acetylneuraminic acid, a member of the sialic acid family, is added to terminal galactose or N-acetyl galactosamine residues which cap glycan chains (**Figure 3** **).** If the galactose residue is not capped, then the galactose is recognized by a lectin in the liver, the asialoglycoprotein receptor (ASGPR) also known as “The Ashwell–Morell Lectin” (44, 45). ASGPR clears desialylated glycoproteins with exposed non-reducing D-galactose (Gal) or N-acetylgalactosamine (GalNAc) as end groups.

The complexity of the surveillance by the ASPGR is not yet fully understood. For example it may seem counterintuitive but it has been shown that ASGPR clears glycoconjugates terminating with sialic acid alpha-2,6 GalNAc (45, 46). That linkage is absent in recombinant FSH produced by CHO-cells but is present in human glycoproteins. The alpha-2,6 linkage is also present when follitropin is expressed in cells of human origin. Therefore in that case, addition of sialic acid alpha-2,6 hastens removal of serum glycoproteins (47, 48) despite capping galactose. The implications are that the presence of the positional placement of the alpha-2,6 sialic acid will not likely serve to deter clearance. It does demonstrate for the purpose of this review that more charge does not necessarily translate into prolonged half-life. In fact, the wrong charge (ie. sialic acid alpha-2,6 GalNAc) may enhance clearance. This then adds to the complexity of understanding charge in relation to clearance and will likely affect the pharmacokinetics of glycoprotein biopharmaceuticals manufactured using human cell lines which add sialic acid alpha-2,6GalNAc.

In humans, expression of the hepatic ASGPR is lower than in rodents, suggesting that dependence on this clearance mechanism in humans is limited (49). However, if the rodent is used as the bioassay for comparisons of bioactivity in *in vivo* and a preparation has a high sialic acid alpha-2,6 GalNAc content, the potency estimates will not accurately reflect potency in humans. Since both alpha-2,3 sialylation and alpha-2,6 sialylation occurs in humans (50) an open question is whether variations in their occurrence in manufactured follitropin will significantly affect the clearance rates. A meta-analysis which included both recombinant FSH and HP-FSH (<0.1 IU LH) illustrated that such differences between FSH preparations may in the end balance out (51). If one accepts that gonadotropin amount per oocyte is a biopotency measure, there was no difference in outcomes at the same dose. Those results suggested that, although recombinant FSH (less acidic and with only sialic acid alpha-2,3 GalNAc) and HP-FSH (more acidic and with both sialic acid alpha-2,3 GalNAc and sialic acid alpha-2,6 GalNAc) differ in isoform composition and charge these gonadotrophins have a comparable *in vivo* efficacy in terms of clinical pregnancy. It is worth noting that in a previous meta-analysis some end points associated with direct effects of follitropin on number of follicles on the day of hCG administration, number of oocytes acquired, and duration of treatment as well as amount of ampules/women, favored recombinant follitropin, but with no differences in pregnancy outcome (54). The urinary gonadotropins are more acidic and should have a longer half-life, so at the same dose should be more potent. However the urinary gonadotropins have the alpha 2,6 sialylation which can bind to ASGPR lectin which could affect its biopotency estimates in the rat and ultimately the amount of protein in the vial per IU.

In summary, the structural attributes of follitropin which determine its *pharmacokinetic* profile rest largely in its *glycosylation* and *sialylation*. Importantly, for maximum circulatory persistence, filling all four of the glycosylation sites is important. Also, completely capping all glycan chains that have potential sialic acid α2,3 sites, such as N-acetyl, 1,4GlcNac and Galactose (**Figure 3A**), with sialic acid α2,3 can provide for maximum circulatory half-life. Finally, the net charge, i.e. isoforms, determined by isoelectric focusing or chromatofocusing is of relatively limited use to ascertain these properties (**Figure 2**). The net charge of a theoretical isoform can be the same for a variety of different glycoforms of follitropin (5). This is because those methods do not separate follitropin based on glycan structure (52). Mass spectrometry has proven more useful to accurately characterize follitropin preparations.

## Structural Aspects of Follitropin which Affect *Pharmacokinetics, Pharmacodynamics*, and Clinical Response in Controlled Ovarian Stimulation (COS) Protocols

*Pharmacodynamics:* Pharmacodynamics of follitropin include its molecular effects at the level of its target cells, the Sertoli cell in the testis and the granulosa cells in the ovary, mediated by receptor binding, recycling, and post receptor binding effects (53). These effects are underpinned by production of intracellular second messengers, activation of the phosphokinome and transcriptional activation. Historically, the net effects of most interest to follitropin have been the production of secretagogues, particularly the sex steroids estrogen and progesterone, and cellular proliferation. With respect to proliferation, efficacy of induction and maintenance of high-quality spermatogenesis would be the measure in the male. Growth and maturation of preovulatory follicles and high-quality oocytes is the desired therapeutic effect in the female. Here, it is critically important that the oocytes will yield high quality blastocysts following *in vitro* fertilization. The next section of the discussion will focus primarily on how structural differences in follitropin can or has been demonstrated to differentiate its pharmacodynamic properties.

*Sialylation:* In the previous section the structural differences in sialylation were discussed in terms of circulatory half-life. Follitropin half-life will affect efficacy and depending on glycosylation status, may or may not require special dosing regimens. CHO cells glycosylation machinery is very similar to that found in human cells but with two major differences: they lack a functional acetyl-glucosaminyl transferase- III (GnTIII) for the addition of bisecting-GlcNAc and, more importantly, they lack the alpha-2,6-sialyltransferase-I activity (ST6Gal-I or SIAT1) responsible for the addition of sialic acid apha-2,6 on galactose residues (54). In studies sponsored by the manufacturer of Follitropin Delta (Rekovelle^®^), already approved in several countries including those from the European Union, Canada, Australia and some from Latin America) the sialylation differences were reported to affect both follitropin pharmacokinetics as well as pharmacodynamics (55, 56). In women, but not in rats, the pharmacokinetics favored Follitropin Delta produced using a human embryonic retinal cell line (PER.C6^®^), compared to the reference follitropin preparation Gonal-F^®^, expressed by CHO cell lines (55, 56). Differences between the pharmacokinetics in humans and rodents could be due to the lower expression of the asialoglycoprotein receptor in the former (48). Interestingly, the *in vitro* bioactivity of both preparations were comparable (57), a finding that contrasted with previous studies comparing follitropin alpha and urinary FSH (58), which exhibit distinct degrees of sialylation. It also seemed surprising that follitropin Epsilon exhibited more favorable pharmacodynamic properties than urinary FSH (59), given that both are of human origin and bear sialic acid residues at positions α2,3 and α2,6. Differences in complexity of glycans and amount of sialic acid residues, which may differentially impact on the activation and signaling at the human FSHR might explain the different pharmacodynamic effects of both preparations.

Unfortunately, the field does not require a written description of the product regarding all biochemical attributes. Unless a study is performed to independently determine the carbohydrate profile of these preparations as well as the batch-to-batch consistency, that information would not be publicly available. One simple explanation for the differences found between Rekovelle^®^ and the follitropin alpha preparation may be due to the potency estimates of their content, which are based on an *in vivo* bioassay in rats. Thus, despite administration of identical bioactive doses to women, (expressed as international units [IU] based on the Steelman-Pohley *in vivo* rat assay) of Gonal-F^®^ and Rekovelle^®^ the data revealed *slower* clearance for Rekovelle^®^ and significantly higher pharmacodynamics responses of this preparation in terms of serum E2 and inhibin, as well as number and size of follicles (56). This seemed remarkable because the patent discloses that a α2,6 sialic acid preparation of follitropin had a *drastically reduced half-life compared* to the α2,3 sialic acid follitropin prepared in the same cells (60). The cell line used was prepared by subjecting the parental cell line to an engineering step with the addition of the gene encoding for the α2,6-sialyl-transferase. The resulting follitropin was highly sialylated showing sialic acid content and isoform distribution comparable with urinary follitropin from postmenopausal women. However, the material was *cleared very rapidly from circulation of rats at a rate comparable to the original material which had lower sialic acid content* (60). This was an unexpected observation since it is known that a proportion of sialic acid on natural and biologically active follitropin is α2,6-linked, but is consistent with the discussion above. Not surprisingly, the clearance of the α2,6-sialylated follitropin was found to be mediated by the ASGPR found in the liver. This was demonstrated by transient blockade of the ASGPRs using an excess of another substrate for the receptor (57, 60). Therefore, it seems reasonable to assume that the reason why the α2,6 and α2,3 sialic acid follitropin was more potent than the α2,3 sialic acid follitropin comparator in the pharmacodynamic studies (56), was due to the fact that the dosing was based on the *in vivo* bioactivity determined in a rat bioassay. In that case the α2,6 and α2,3 sialic acid follitropin would have cleared faster, so to achieve the same potency, and then to be dosed based on equal potency, there would have been more mass added in the pharmacokinetic and pharmacodynamics studies. Perhaps additional studies with the α2,6 and α2,3 sialic acid follitropin *based on mass* are needed to evaluate if there is indeed a greater efficacy with respect to those parameters.

It should be noted that Rekovelle^®^ is dosed by mass (micrograms) not in IU since the rat bioassay might not fully reflect the potency of the FSH in Rekovelle^®^ in humans. The dosing regimen is specific for Rekovelle^®^ and the microgram dose cannot be applied to other gonadotropins, so it is not an interchangeable biosimilar. For the first treatment cycle, the individual daily dose is to be determined based on the woman’s serum anti-Müllerian hormone (AMH) concentration and her body weight. In a multicenter study of Rekovelle^®^ to test the dose relationship to ovarian response, increasing doses *based on mass* induced a yield of oocytes (primary endpoint), roughly 1 oocyte per microgram of Rekovelle^®^. Remarkably, there was no dose relationship of the yield of good quality blastocysts (a secondary endpoint) (61). Those data suggested that oocyte yield as a goal of controlled ovarian hyperstimulation might not be the best target. Thus, for each round of stimulation there will be a finite pool of oocytes that yield high quality blastocysts. The study included Gonal-F^®^ at the midrange dose but without an intention to use this preparation as a comparator. Thus, it was not possible to test the hypothesis that the “more human” follitropin (that is, follitropin with an additional sialic acid at position α2,6) is better at recruiting follicles that will yield high quality blastocysts. For almost all doses of Rekovelle^®^, however, the yield of high-quality blastocysts was higher if a patient had higher anti-Müllerian hormone (AMH) levels. Accordingly, at face value, Rekovelle^®^ did not offer a therapeutic advantage in eliminating the ovarian reserve gap between women with high and low AMH. A further, non-inferiority, multi-center trial (ESTHER-1) yielded similar results in terms of the co-primary endpoints. For example, similar results for ongoing pregnancy rate, ongoing implantation rate, and quantity and quality of oocytes retrieved were observed. These results had no net differences between follitropin delta dose, adjusted to anti-Müllerian hormone AMH levels, and the conventional, non-AMH adjusted Gonal-F^®^ dose as per international recommendations (62). Noteworthy, however, AMH stratification led to a modestly lower incidence of women with poor response as well as fewer instances of ovarian hyperstimulation syndrome (OHSS) of any grade (particularly in women with high baseline AMH levels), compared with conventional follitropin alpha treatment. Few advantages were, therefore, found for follitropin delta over follitropin alpha. Nevertheless, the observation of moderate/severe OHSS risk and less need for preventive interventions, particularly in women within the highest AMH quartile and whenever AMH was used to define the dose of the former (later confirmed by the ESTHER-2 trial) may be considered to be an advantage (63, 64). Further, in all these studies, doses of follitropin delta were fixed, whereas for the comparator, dose was based on international recommendations and the individual criteria of the treating physician, so that a vis-à-vis comparison is even more difficult due to these flexible adjustment criteria to determine the follitropin alpha dose. Interestingly, a post-hoc analysis of those two multicenter studies (65) concluded that doses of 10 micrograms follitropin delta [the pre-maximal dose employed in the first study (61)] and the conventional 150 IU of follitropin-alpha led to similar ovarian responses [for further discussion see refs (66, 67)].

Particularly because of the concerns that there is a significant negative correlation between age and IVF success (68), a goal should be to generate more viable blastocysts per cycle. This may be achievable by delaying retrieval until the next cycle which would benefit “conditioning” of the follicle pool. It would serve women better cost-wise if a protocol could be developed within the same cycle with care taken not to promote endometrial advancement, which can lead to decreased pregnancy rate (69). Perhaps it would be possible to develop a glycoform of follitropin which would increase AMH to promote preantral follicle growth, then switch to a follitropin that also induces antral follicle maturation and recruitment (70). Nevertheless, in regard to this review, and with regard to the question at hand, the dose of FSH needed to produce viable blastocysts and embryos does not seem to advocate for forms of follitropin which are marginally better than the reference since the cohort of follicles that will produce oocytes/blastocysts of high quality is limited and recruitment of additional follicles is not productive with regard to high quality oocyte and blastocyst yield (61). As mentioned above (62) only a small number of oocytes per retrieval can develop into a competent blastocyst/embryo. Neither study was designed, nor is it possible to determine if there is a difference compared to the reference. Thus, it is not clear if the desired pharmacodynamic effect of high-quality blastocysts was significantly better for the more “human” follitropin. However, one must wonder if there is an added efficacy of the α2,6 and α2,3 sialic acid follitropin because the α2,6 sialic acid seems to compromise longevity of circulation. Teleologically speaking one must assume that the unique human sialylation has some role. It is not possible to know if the ratio of Rekovelle^®^ glycoforms is similar to the reference preparation Gonal-F^®^ because that information is not publicly available, albeit it is known that Gonal-F^®^ is primarily bi-glycosylated (9) (**Figure 1**). Without such information it is not possible to attribute the longer half-life to any particular structural feature, particularly since the inventors already demonstrated that α2,6 sialic acid *only* follitropin was cleared more rapidly than any other preparation of follitropin. Indeed, the end game is high quality oocytes/blastocysts, and the data from those prospective trials suggest that more individualized gonadotropin-dosing regimens may be necessary and sufficient to achieve desired outcomes (61). If that is the case, then there will be a need to better define the gold standard of desired pharmacodynamics effect and efficacy.

The preparation of a second “human-like” preparation of follitropin also has recently been published (follitropin epsilon^®^, Glycotope^®^, Germany; currently not marketed). This is put in a category of follitropin epsilon, to distinguish it from Reckovelle^®^ even though a human cell line is also used for biomanufacturing of follitropin epsilon (the HEK293 based expression system GlycoExpress^®^) (71). A single-dose as well as multiple-dose administration was used and compared to recombinant CHO cells-derived Gonal-F^®^ as well as urinary derived follitropin (Bravelle^®^). The doses were based on the Steelman Pohley bioassay. Overall, follitropin epsilon, represented as having a fully human glycosylation, showed a comparable pharmacokinetic profile to Gonal-F^®^ (71). This is markedly different from the report of Rekovelle^®^ which evidenced a longer half-life than the reference Gonal-F^®^ (55, 56). Despite the similar pharmacokinetic profile, the pharmacodynamic properties (follicle growth, serum inhibin B, and serum E2) differed between products, favoring follitropin epsilon. It was remarkable that follitropin epsilon induced a peak E2 of around 150 pg/ml whereas both urinary and recombinant follitropin did not reach 40 pg/ml. Here again, one possible explanation for the disparity in E2 production is that bioassay estimates from the rat bioassay do not accurately translate to the human. In that case if dose was based on IU, then there would be a difference in mass injected. An alternative explanation would be that the differences in glycosylation are affecting pharmacodynamics at the level of the ovarian follicle. That this is plausible has been demonstrated with FSHR antagonist (ADX68693) which augments receptor binding and steroid production (72). If that were the case then pharmacokinetics which are not different would have no effect. Accordingly, the authors do posit that differences seen for both Rekovelle^®^ and follitropin epsilon molecules may indicate that the human glycosylation patterns present on these molecules play an important role in their action on the FSH-receptor, and if true, then in regard to this review, their study as to how this may be occurring could lead to advances in understanding of ovarian physiology and particularly post-binding sequelae.

A more recent phase II multicenter study analyzed different doses (in IUs) of follitropin epsilon and the standard gonadotropin alpha dose (i.e. 150 IUs) on the number of follicles sized ≥12 mm (primary outcome), oocyte number, and hormone concentrations (73). Regarding the primary outcome, modest differences favored follitropin epsilon over follitropin alpha at equivalent doses (150 IU). Interestingly, the superior pharmacodynamic properties of follitropin epsilon *vs* follitropin alpha in terms of serum E2 and inhibin-B were not reproduced in magnitude compared to the first study (71, 73), thus underlining potential batch-to-batch differences in potency among the gonadotropin alpha ampules employed in these studies. Also interesting was the finding that at equivalent doses, follitropin alpha was superior in terms of important secondary outcomes, including clinical pregnancies and live births. Differences in other outcomes between follitropin epsilon and follitropin alpha are, as discussed earlier, difficult to explain due to the *in vivo* bioassay employed for the dose calibration of the preparations.

Unfortunately, without a clear, detailed, side-by-side biochemical analysis of these hormone preparations, it cannot be ascertained whether the differences observed are due to differences in production choice of cell lines or differences in potency determination. It is difficult to make any sense of the data, no matter how intriguing, and conclude whether carbohydrate *structure* makes a difference in pharmacodynamics. However, it raises an important issue that seems to be unique to biopharmaceuticals, and particularly important to the gonadotropins: What is the best way to determine potency?

Clearly there appear to be issues with *in vivo* bioassays particularly with glycoprotein glycosylation variation and differences in sialylation and clearance of glycoproteins between species. For the gonadotropins, there seems to be a need for more dosing studies, particularly with conventional follitropin preparations, which should be based on mass and done in humans. Of course, the human subjects must fit strict criteria for the comparisons and such comparisons may not be feasible due to costs. Alternatively, cost benefits can be based on protein in the vial or pen. Then it becomes a matter of which preparation is better? Amino acid analysis to determine protein content of glycoproteins preparations have suffered from interference by Maillard reactions. These yield a residue which prevent accurate recovery of amino acids. However this issue has been resolved using gas-phase hydrolysis and reversed phase HPLC (74). Alternatively, protein concentration can be determined by instituting an agreed upon extinction coefficient and measurement at A^280^. Only then can the effect of carbohydrate given its complexity be accurately assessed *in vivo* and given the price point based on protein mass, patients will decide the better value. Insights about activity at the level of the ovary can, on the other hand, be gained from additional *in vitro* studies. These comparisons cannot be completely understood without comprehensive analysis of the nature of glycan attachment as well.

**Figure 4:**
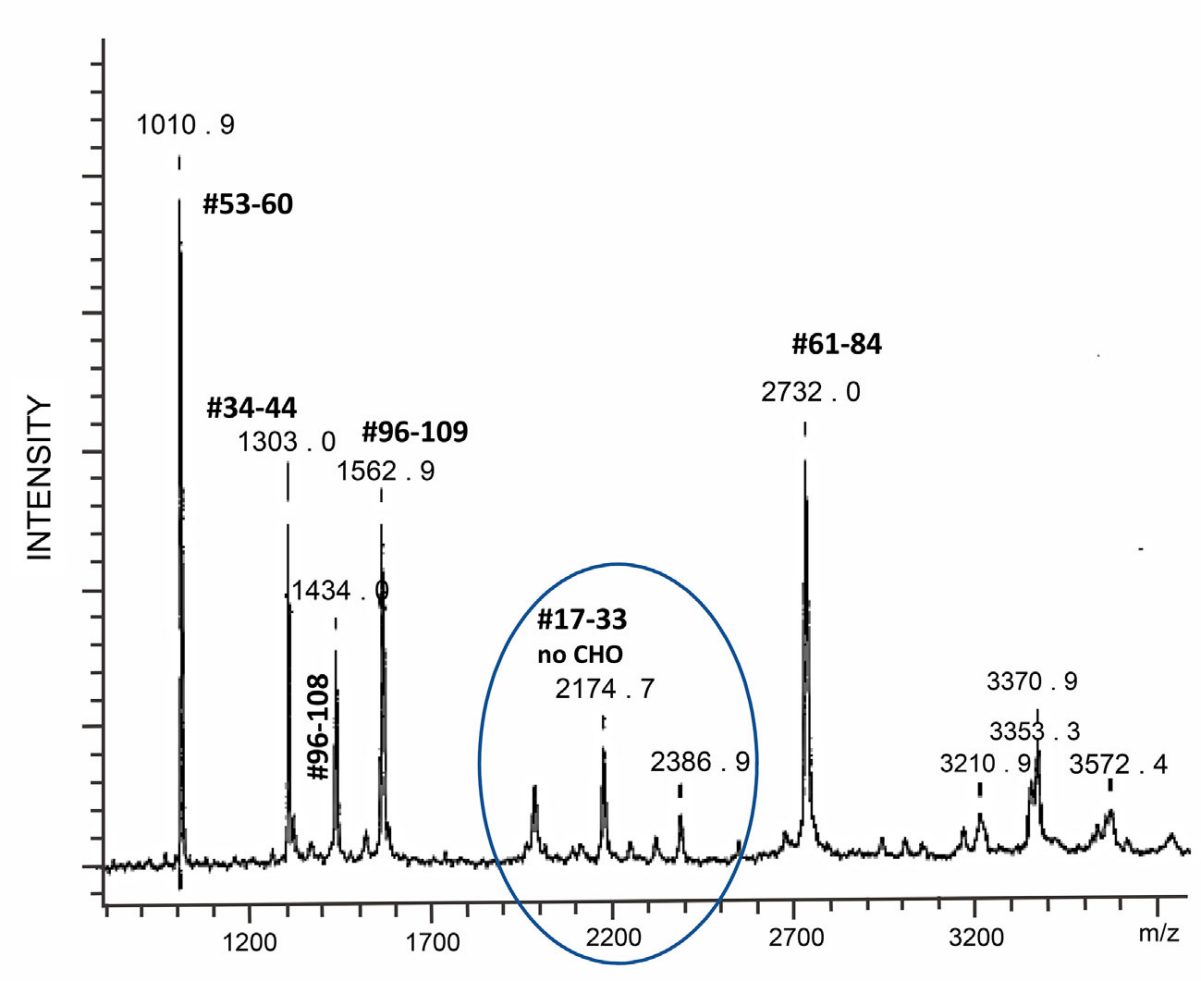
Mass spectrometry tryptic map of an hFSH beta subunit expressed in insect cells. One of two possible N-linked glycosylation sites is not used with high fidelity as evidenced by the presence of peptide #17–33, whose molecular ion is equivalent to the peptide hFSHbeta 17–33 mass without glycosylation. From (37) with permission. Tryptic digestion of human FSH produced in insect cells revealed a loss of glycosylation at asparagine 24 demonstrating that this glycosylation site is not consistently glycosylated.

*Hypoglycosylation:* When human follitropin is expressed in insect cells, it is made as a high mannose glycoprotein (36). It is comparable to fully glycosylated human pituitary follitropin in activity at the level of the receptor and induces cAMP and steroid hormone production (75). These properties provided for a systematic analysis of the structure and function of human follitropin using insect cell expressed human follitropin and alanine scanning mutagenesis (36, 76). In the course of production, purification and crystallization trials of insect cell derived follitropin, mass spectrometry revealed a single tryptic peptide with an atomic mass of 2174.7. This peptide corresponded to follitropin beta residues 17 to 33 without a carbohydrate attached (**Figure 4**) (37). The realization that this site was not consistently filled, encouraged the preparation of a mutant form of follitropin that could not glycosylate at that site, and resulted in preparation of diffraction quality crystals of glycosylated follitropin and solution of the crystal structure of human follitropin that was glycosylated at three sites (36). This glycoform of follitropin was fully active and diffracted to 3.0 Å. Seven glycan residues (two at N52α, three at N78α and two at N7β) have been included in the final refinement (35). Comparative analysis of follitropin and hCG structures indicated that glycosylation has no global effect on the glycoprotein hormones conformations.

Carbohydrate does not participate in the primary binding of follitropin to the follitropin receptor monomeric hormone binding domain (19). However, in the trimeric structure of the follitropin receptor extracellular domain, which includes the hormone binding domain, it is not possible to dock more than one fully glycosylated follitropin molecule on the trimeric structure, suggesting that carbohydrate and in particular the glycan at αN52 may facilitate the dispersion of follitropin receptor into dimers and/or monomers (17).

Substantial conformational changes in several regions of follitropin were revealed upon superimposition of two follitropin heterodimers in the unit cell of free glycosylated follitropin (17). In regard to the beta subunit region which contains the two glycosylation sites, E16 to R18 in loop L1β, K40 to Q48 in loop L2β are included in all noted conformational changes which collectively result in a Cα root mean square deviation (r.m.s.d) of 1.9 Å between the two follitropin copies. The largest shift is over 5 Å for the residues in loop L2β and COOH-terminus of both chains. In contrast, the follitropin conformation in the follitropin receptor-bound form is quite rigid. In addition to others, K14 in loop L1β, Y39, K40, and P45 in loop L2β show some minimal movement (17). Whether this is because the crystal of the FSH-FSHR complex was deglycosylated prior to diffraction is not clear. Tentatively, the interpretation is that the significantly reduced r.m.s.d. implies that follitropin rigidifies its overall conformations upon receptor binding. Given that hypoglycosylation occurs primarily due to β-subunit hypoglycosylation, and in particular at βN24 (see below), it leads one to speculate that conformational flexibility and long-range interactions, potentially quaternary stabilization and flexibility may be affected by absence of carbohydrate at that locus. The crystal structure of free follitropin is devoid of carbohydrate at that loci, by design. The crystal structures of the follitropin – hormone binding domain, or the follitropin – full extracellular domain also lack carbohydrate at this locus following endoglycosidase treatment. Thus, it is an open question whether glycosylation of the β-subunit affects the structure of follitropin and yet it has clearly been shown to affect its pharmacodynamics (9, 77). Moreover it does appear that hypoglycosylated FSH has greater accessibility to FSHR or causes structural shifts in FSHR that unveil additional binding sites (77). FSHR appears to be distributed in various oligomeric forms at the plasma membrane (59) as is LHR (78) and the functional significance of these forms has been studied extensively by a variety of methods (79). It is quite likely that not only initial binding events but also intracellular trafficking and persistent signaling (80) could be affected in different ways by different glycoforms by virtue of changing structural attributes of the hormone receptor complex.

## Naturally Occurring Glycoforms: Challenging the Future of Follitropin Administration in the Clinical Arena

As previously discussed, the heterogeneity of gonadotropins stems largely from their carbohydrate complexity (8, 37), and it is well documented that pituitary gonadotropins are secreted into the circulation as a mixture of variants, which are virtually identical in primary structure but are different in regard to oligosaccharides attached to the protein core. The oligosaccharide structure confers distinctly different physiological features such as plasma half-life and potency at the target cell level (38, 81). As previously mentioned, oligosaccharide heterogeneity exists in two forms, microheterogeneity, which results from variation in the type of carbohydrates comprising the oligosaccharides attached to the protein core (38), and macroheterogeneity, which comes from the absence of one or more oligosaccharide chains from a hormone variant (11).

**Figure 5:**
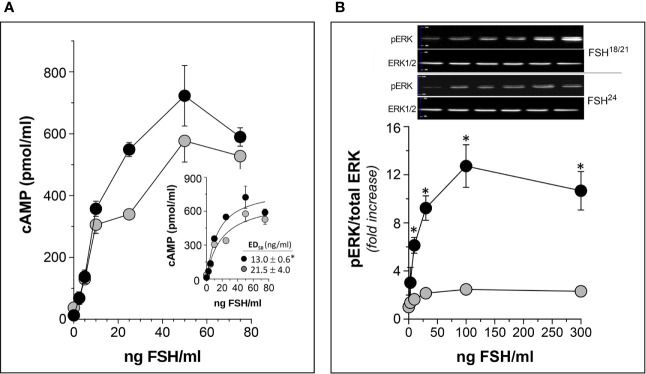
*In vitro* potency of human follitropin glycoforms FSH^18/21^ and FSH^24^ as disclosed by two different bioassays in HEK293 cells stably expressing the human FSHR. **(A)** Dose-response curves of total (intra- *plus* extra-cellular) cAMP production by cells exposed to FSH^18/21^ and FSH^24^ for 3 h. *Inset:* non-linear regression curve of data shown in the main graph with the corresponding ED_50_ values of each glycoform (*p<0.05 FSH^18/21^ *vs* FSH^24^). **(B)** Dose-response curves for ERK phosphorylation stimulated by increasing concentrations of FSH^18/21^ and FSH^24^. Representative immunoblots from a single experiment are shown at the top of the graph. *p < 0.03 FSH^18/21^ *vs* FSH^24^ at 10, 30, 100, and 300 ng/ml FSH doses. Data are presented as mean ± SD from three independent experiments. Modified from (9), with permission.

As described above, human FSHβ subunit is glycosylated at Asn^7^ and Asn^24^ residues, and differences in the number of glycans on this subunit (none, one or two) constitutes the basis of the *macroheterogeneity* of the α/β dimer (7). (**Figure 1**). *In vitro*, *in vivo*, and *in silico* studies have shown that hypo-glycosylated pituitary human FSH preparations exhibit shorter plasma half-life but higher FSH receptor (FSHR) binding activity and *in vitro* bioactivity as compared to fully glycosylated FSH (9, 32, 77, 82, 83) (**Figure 5**). Thus, like the influence of microheterogeneity on the capacity of the gonadotropins to activate intracellular signaling (38), it seems that the extent of glycosylation of the FSHβ subunit determines not only its pharmacokinetics but also its pharmacodynamic post-binding bioactivity. Differences in FSH glycosylation appear to influence the stability of binding to the FSHR, with a more stable FSH/FSHR interaction of the hypo-glycosylated FSH glycoform relative to the fully-glycosylated FSH. Differences in FSH glycoform binding suggest mechanisms for the variant and differential biological effects of fully and partially glycosylated FSH *in vivo* and *in vitro* (9, 32, 77, 84).

**Figure 6:**
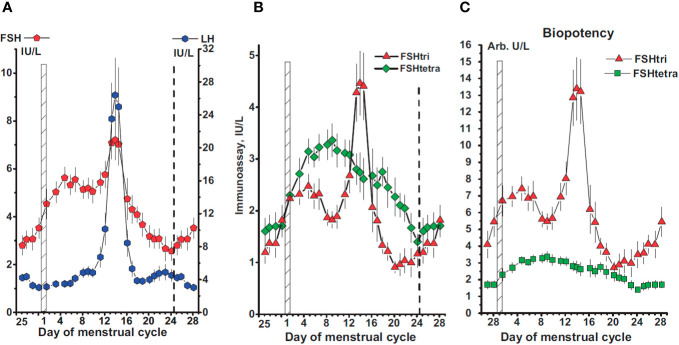
**(A)** Concentrations of FSH and LH in serum samples from 78 women with a normal menstrual cycle. The day of the menstrual cycle is given and the first day indicated by a vertical hatched bar. The ovarian cycle starts on day 25 of the previous cycle and lasts to day 24 of the menstrual cycle, the end indicated by a vertical dashed line. **(B, C)** Concentrations of FSH-tri and FSH-tetra **(B)**, and their estimated biopotencies **(C)**, in arbitrary units per L, during the normal menstrual cycle. Data in this figure are plotted as three-day moving mean values. Reproduced from (86) with permission.

Macroheterogeneity of pituitary FSH is important from the physiological point of view given that recent evidence has shown that fully-glycosylated FSH represents approximately 80% of FSH in pooled pituitary and urinary FSH samples from postmenopausal women, while partially glycosylated FSH represents 52%–70% of the samples isolated from pituitaries derived from autopsies of women in reproductive age (7, 85). Further, the abundance of the low molecular weight glycoform, FSH^21^, is correlated with the age of the donor; the FSH^21^ glycoform is more abundant in pituitaries of younger women and decreases over the reproductive lifespan, whereas, in postmenopausal women FSH^24^ is the dominant glycoform (57). Recent studies of circulating gonadotropins also suggest that the levels of hypo- and fully-glycosylated FSH and LH vary across the menstrual cycle (**Figure 6**), with higher levels of fully-glycosylated FSH than hypoglycosylated FSH during the follicular phase and luteal phase and the inverse during midcycle (**Figure 6B**), suggesting that the hormonal control of glycosylation depends on the physiological status of the donor (86). If eventually macroheterogeneity proves to be biologically important from a regulatory standpoint, it should be possible to design new recombinant preparations containing varying proportions of fully- and hypo-glycosylated follitropin that may be adjusted depending on the particular characteristics of the patient and day of stimulation, allowing more physiological control over follicular maturation and development of predominantly high-quality oocytes, with less secondary effects.

## Conclusions and Future Perspectives

Clinical studies that assess pharmacokinetic and pharmacodynamic similarity are important components of a demonstration of FSH biosimilarity particularly if one preparation is to substitute for another within an assisted reproduction treatment cycle (16). Biosimilars may provide financial savings to patients and healthcare plans in much the same way as a generic drug and may open the market to more innovation in modality of delivery, etc. The case of FSH biosimilars is nuanced by the potential differences in carbohydrate, which may not affect pharmacodynamic properties of early signal transduction pathways and other canonical readout of biological function. Pharmacodynamics which are more difficult to measure, such as persistent signaling, receptor parsing in early and very early endosomes, and recycling could potentially be affected more by the impact of carbohydrate variations on hormone receptor complexes oligomeric nature including flexibility, stability and interaction with adapter and effector proteins (78, 80). Thus, if a difference in glycosylation may affect pharmacokinetics as well as pharmacodynamics, these differences may not be detectable in the traditional *in vitro* assays. These may be more strategically studied by more accessible methods such as gene expression and phosphoproteomics and in rested primary cultures of human granulosa cells (87). Moreover, patient response is so varied and complex, and doses of FSH so high and so varied between patients in the clinical practice that such subtle differences while of interest from a basic science perspective may be obscured. Additionally, impacts on the subsequent cycles of IVF may reveal additional information and may be relevant to emerging practices such as luteal phase stimulation (88–90), despite its controversy (91), and confusion over nomenclature (92).

Although a higher bar of purity and consistency is set for advancements of next generation FSH preparations, improving on the “physiological milieu” of glycoforms is a worthy endeavor. Commercially available preparations of FSH that have intentional modifications to prolong half-life are possible and corifollitropin alfa^®^ is one such example (26). Ironically, in the endeavor to reach the high bar, the physiological is subverted. That is because glycosylation of the naturally occurring FSH secreted from the pituitary is complex and varies with age and menstrual cycle stage (86, 93). Capitalizing on this observation, attempts are underway to specifically and intentionally prepare recombinant preparations of FSH that represent FSH glycoforms predominantly observed in younger women (77). Thus, development of future preparations of FSH may focus on matching the most active FSH forms with the least responsive of patients for which the originator or closely matched “biosimilars” may not optimally induce follicle growth and high-quality oocytes. This may ameliorate the conundrum of whether or not to increase the dose of FSH in patients that are low responders (94). Moreover, hypoglycosylated FSH which may have shorter half-life could afford greater control over hyperstimulation by virtue of faster clearance and easier dose control.

Glycoprotein therapeutics such as FSH, while providing challenges with regard to achieving identical glycosylation batch-to-batch, are rarely evaluated on that basis. Sialylation can clearly affect pharmacokinetics and by now it should be clear that charge differences should not be part of the lexicon for quality control or to classify gonadotropin preparations. Advanced methods such as mass spectrometry provide a somewhat clearer yet imperfect picture. Complexity of the antennary structures adds further complexity. Poorly defined roles of carbohydrate on structural flexibility and the role of the same on oligomerization and post-binding endosomal sorting of the receptor is a gap. Additionally, little is understood of the consequence of fucosylation, even though it has been shown to affect flexibility of immunoglobulin by altering the protein backbone interactions. Embracing this complexity, it seems safe to summarize by acknowledging that structural differences in carbohydrate affect biological function and clinical effect. Type and completeness of sialylation will affect pharmacokinetics certainly and potentially pharmacodynamics. Glycan structures can contribute to carbohydrate-protein and/or carbohydrate-carbohydrate interactions and may function as “molecular glue” to help stabilize inter- and intra-molecular interactions. More mobile N-glycans the electron density of which is usually missing on X-ray crystallography, may guide FSH to its receptor binding site and impact oligomerization and/or adapter and effector sites away from the ligand-binding site. Finally, specifically with regard to the glycosylation of FSHβ subunit, a hypothesis is that its conformation is dependent upon glycosylation and may vary with the same. This is played out in a variety of established pharmacodynamic effects in mouse models (33).

## Author Contributions

Both authors contributed to the article and approved the submitted version.

## Funding

Studies performed in the authors’ laboratories have been supported by the Consejo Nacional de Ciencia y Tecnología (CONACyT) (grant 240619), and the National University of Mexico (UNAM) (to AU-A).

## Conflict of Interest

The authors declare that the research was conducted in the absence of any commercial or financial relationships that could be construed as a potential conflict of interest.
